# Seroprevalence and risk factors of tick-borne encephalitis in Mongolia between 2016 and 2022

**DOI:** 10.1016/j.parepi.2023.e00318

**Published:** 2023-07-24

**Authors:** Dashdavaa Ganbold, Bayarsaikhan Uudus, Naranbat Nyamdavaa, Yeruult Chultemsuren, Amarbayasgalan Zagd, Mungunzaya Tangad, Burmaa Badrakh, Bolorchimeg Baldandorj, Ochgerel Dogsom, Rolomjav Lkunrev, Uyanga Baasandagva, Tsogbadrakh Nyamdorj, Narankhajid Myadagsuren

**Affiliations:** a Department of Biology, School of Biomedicine, Mongolian National University of Medical Sciences, Zorig Street 3, 14210 Ulaanbaatar, Mongolia; b Department of Biology, School of Sciences and Art Science, National University of Mongolia, Zaluuchuud Avenue 1, 14201 Ulaanbaatar, Mongolia; c Gyals Medical Center, Peace Avenue-61A, Bayangol, 210351 Ulaanbaatar, Mongolia; d Department of Pharmacology, School of Biomedicine, Mongolian National University of Medical Sciences, S. Zorig Street 3, 14210 Ulaanbaatar, Mongolia; e Department of Cardiology, School of Medicine, Mongolian National University of Medical Sciences, S. Zorig Street 3, 14210 Ulaanbaatar, Mongolia; f Department of Laboratory, National Center of Mental Health, Bayarzurkh District, IX Khoroo, Sharkhad, 13020 Ulaanbaatar, Mongolia; g Department of Obstetrics and Gynecology, School of Medicine, Mongolian National University of Medical Sciences, S. Zorig Street 3, 14210 Ulaanbaatar, Mongolia; h National Center for Zoonotic Disease, Songinokhairkhan District, 20 Khoroo, 18131 Ulaanbaatar, Mongolia; i Department of Biology, School of Biomedicine, Mongolian National University of Medical Sciences, S. Zorig Street 3, 14210 Ulaanbaatar, Mongolia

**Keywords:** Tick-borne encephalitis, Seroprevalence, Risk factor, Mongolia

## Abstract

The tick-borne encephalitis virus (TBEV) is a zoonotic agent that causes severe encephalitis in humans and is transmitted through the bites of infected ticks. *Ixodes* ticks are the primary vector for TBEV in Mongolia, and approximately 3.4% carry the TBEV. The ticks are capable of not only transmitting these viruses but also serve as excellent reservoir hosts. The *Dermacenter* tick species may have similar properties. TBEV is a significant cause of virus-related diseases of the central nervous system in many European countries as well as in China, Russia, and Mongolia. Our objectives were to investigate TBEV seroprevalence and infection risk factors in different biogeographical zones and provinces, especially in the highly endemic areas of Mongolia. Serum samples were collected from individuals who experienced tick bites (*n* = 993) in Mongolia between 2016 and 2022. Enzyme-linked immunosorbent assay of the samples was performed to evaluate for TBEV-specific immunoglobulin (Ig)M and IgG. We analyzed the risk factors and seroprevalence of TBEV infection among these individuals using a cross-sectional, questionnaire-based study. Statistical analyses were performed using a multistage cluster sampling survey design, and all data were analyzed using the R software. TBEV IgM and IgG antibodies were detected in 8.1% (80/993) and 20.2% (201/993) of all serum samples, respectively. The seroprevalence was significantly higher in men (68%, 95% confidence interval [CI]: 1.63–3.13, odds ratio [OR]: 2.25) than in women (*p* < 0.001). Additionally, the seroprevalence was significantly higher among unemployed (35.0%, 95% CI: 0.31–0.84, OR: 0.51) than employed individuals (p < 0.001). The seroprevalence was the highest among the 25–29 and 35–39-year age groups (11%, 95% CI: 1.29–5.51, OR: 2.65 and 11%, 95% CI: 0.94–3.87, OR: 1.9, respectively), and the lowest in the 65–69-year age group (4%, 95% CI: 0.46–6.15, OR: 1.83) (p < 0.001). Furthermore, the seroprevalence was the highest in Selenge province and the capital city Ulaanbaatar (40%, 95% CI: 1.73–21.7, OR: 5.07 and 28%, 95% CI: 0.51–6.89, OR: 1.57, respectively) and the lowest in Bayan-Ulgii and Dornod provinces (0.5%, 95% CI: 0.06–12.4, OR: 1.33 and 0.5%, 95% CI: 0.03–6.24, OR: 0.72, respectively). TBEV infection incidence remained low in most regions of Mongolia but increased in endemic areas. Furthermore, in the univariate subgroup analysis, age, occupation status, and residential area were significantly associated with TBEV seroprevalence.

## Highlights

• Tick-borne encephalitis virus infects more males than females.
• Viral incidence remains low in most Mongolian regions but is high in endemic areas.
• Occupation, age, and residential area are associated with the virus' seroprevalence.
• In Mongolia, the infection rate is low in older individuals and high in adults.

## 1 Introduction

Tick-borne encephalitis (TBE) is a zoonotic disease caused by the tick-borne encephalitis virus (TBEV) of genus *Flavivirus* within the *Flaviviridae* family (Ecker et al., 1999). TBEV has been classified into five distinct subtypes based on genetic analysis and geographic distribution: European, Far Eastern, Siberian, Baikalian, and Himalayan (Ecker et al., 1999; Dai et al., 2018; Kovalev and Mukhacheva, 2017). Furthermore, TBE is endemic in western, eastern, and central Europe and in many countries in Asia, including Mongolia (Frey et al., 2012; Muto et al., 2015; Damdindorj et al., 2021). In Mongolia, the first case of TBE was diagnosed and documented in 1989, with the natural foci in Bugant, Khuder, and Zelter districts of Selenge province (Damdindorj et al., 2021). Moreover, in Mongolia, the Far Eastern subtype was isolated from fatal cases (Khasnatinov et al., 2010a; Baasandavga et al., 2017) and the Siberian subtype from those bitten by *Ixodes persulcatus (*Frey et al., *2012**;*Muto et al., *2015**)*.

Approximately 10,000–12,000 cases of medical TBE are announced worldwide each year; however, this is believed to be far below the actual number (World Health Organization, 2011). Most cases of infection are associated with tick bites during outdoor activities in forested areas (Bušová et al., 2018; Fowler et al., 2013; Ruzek et al., 2019). Between 1998 and 2002, population epidemiological surveillance in Mongolia identified 161 cases of tick-borne infections, with 90 cases linked to TBEV (Adiyasuren et al., 2002). Furthermore, according to the data published by the World Health Organization (WHO) in 2020, encephalitis mortality in Mongolia accounted for 0.18% of deaths, with the age-adjusted mortality rate being 1.16 per 100,000 people, and Mongolia ranks 13th in the world for encephalitis mortality (World health Organization, 2020a). Moreover, in a serological study conducted in 2010, 965 residents of Selenge province revealed a notable finding. Approximately 6.2% of individuals were observed to have specific antibodies against TBEV (Khasnatinov et al., 2010b), where the primary vector, *I. persulcatus*, is prevalent (Frey et al., 2012; Muto et al., 2015).

Among those living in a TBE-endemic area, outdoor activities, specific occupations (hunters, forest workers, and herders), and age are risk factors for TBE (Fowler et al., 2013; Süss et al., 2004; Logar et al., 2006; Krawczuk et al., 2020). However, there have been many cases of infections (80%) occurring in people not considered to be at risk (Zavadska et al., 2013). TBEV, which causes brain inflammation and seizures, is transmitted from the bite of an infected tick to humans (Graus et al., 2016). Although these ticks are not often seen, those living in rural areas may be at a higher risk of the disease than those living in urban areas (Beauté et al., 2018). The symptoms of the disease can be mild-to-severe, and it can be diagnosed in the course of infection, enabling early treatment if appropriate measures are taken (Armangue et al., 2018).

The primary carrier of TBEV is the *Ixodes* tick, which is found in various provinces of Mongolia, such as Selenge, Bulgan, Orkhon, Tuv, and Khentii (Muto et al., 2015). Studies have revealed that 3.4% of these ticks are infected with the TBEV. Additionally, *Dermacenter* ticks serve not only as vectors but also as proficient reservoir hosts for the viruses they carry (Khasnatinov et al., 2010a).

Effective prevention of TBE necessitates knowledge and awareness of risk factors in vulnerable populations as well as knowledge and application of preventions strategies (Lindgren and Gustafson, 2001). Notably, the specific risk factors for TBE include spending time in forests, living near forests, and socioeconomic factors, for example unemployment or employment as a forestry worker (Stefanoff et al., 2012). Additionally, the seroprevalence of TBE has been associated with hunting, tourism, fishing, raw milk consumption (Bušová et al., 2018), skin contact with green spaces, outdoor activity, sex, and age (Ruzek et al., 2019; Danielová et al., 2008; Arnez and Avsic-Zupanc, 2009; Lindquist, 2014).

Between 2008 and 2017, 14 TBE-related deaths were reported in Mongolian hospitals. All 14 patients were reportedly bitten by ticks in Bulgan and Selenge provinces. Additionally, the TBE mortality rates were 28.6% in Bulgan province and 2.7% in Selenge province (Baasandavga et al., 2019). Furthermore, approximately 20 new cases of TBEV infection are reported in Mongolia each year (Khasnatinov et al., 2010a). Notably, a large portion of northern Mongolia is covered with cone-bearing forests, and the southern edge of the Siberian Taiga Forest from Russia runs through the Khangai and Khentii mountains in Selenge and Bulgan provinces in Mongolia. In 2020, health care facilities in Russia received 471,630 calls regarding tick bites, with approximately 25% of the cases involving children and 967 cases being of TBE (0.66 per 100,000 people) (Zlobin et al., 2021). Moreover, Russia experienced an upward trend in the incidence of TBE between 2007 and 2019, with 265 associated deaths (Zhang et al., 2022).

According to a study conducted in 2005, Selenge province had the highest rate of TBEV infection and was the primary endemic area (Adiyasuren et al., 2002; Bata et al., 2005; Bataa et al., 2005). Despite these reports, to the best of our knowledge, there is no study on TBEV infection risk factors in the different biogeographical zones and provinces of Mongolia. TBEV seroprevalence provides the basis for public health recommendations for TBEV infection prevention. Therefore, we investigated TBEV seroprevalence and infection risk factors in different biogeographical zones and provinces, especially in the highly endemic areas of Mongolia.

## 2 Materials and methods

### 2.1 Study area

Mongolia is a country surrounded by land in Central Asia, with Ulaanbaatar as the capital city and a population of approximately 3,409,939 individuals. It shares borders with Russia in the north and the People's Republic of China in the south, east, and west. Additionally, Mongolia's territory of 1,564,116 km^2^ (603,909 mi^2^) is divided into 21 provinces. Although it covers >1,564,116  km^2^, Mongolia is the most sporadically populated country in the world (World Bank, 2019).

Approximately, 34% of Mongolia's population is nomadic, and pastoral herders spend long-drawn-out periods with their livestock. Furthermore, Mongolia is a vast country with mountainous ridges, uneven terrain, and a continental climate resulting in three major regions from north to south: coniferous forests, steppes, and deserts. These areas comprise six natural zones formed by Mongolia's terrain and climate features (Ulziijargal et al., 2020; Temuujin et al., 2022).

1. Alpine tundra zone: This area has a long and cold winter and is the distinctive area above the tree line in the high mountains of Khuvsgul, Khentii, Khangai, Mongol Altai, and Gobi-Altai Mountain ranges, including meadows with scarce vegetation, dense forests, and mountain tops covered with moss. The winter isotherm on these mountain tops is −20 °C in January, and the July isotherm is below 10 °C, with an annual precipitation of >400 mm. Additionally, the vegetative period is short.
2. Boreal forest Taiga zone: This region includes the Mongol and Gobi-Altai Mountain chains, with an altitude of 2000–3000 m (absolute height: 3500–4374 m) above sea level. Moreover, the average annual precipitation is 100–300 mm, and the temperatures in January and July are −30 to-25 °C and 17–18 °C, respectively.
3. Forest steppe zone: This zone is located 1500–2000 m above sea level. The distinctive feature of this biome is the unique combination of steppe and forest elements in alpine areas. Furthermore, the average annual precipitation is 200–300 mm, and the temperatures in January and July are −30 to −25°Cand 15–20 °C, respectively.
4. Steppe zone: Steppe is found 800–1200 m above sea level. This ecozone is a band of grassland, shrub terrain, and mixed forests in northeast Mongolia that follow the course of the Onon and Ulz rivers. This region is covered with grass and is the best example of an undisturbed steppe ecosystem. Additionally, it is one of the remaining areas inhabited by stable herds of large vertebrates in the Paleartic in a semi-mountainous area. The average annual precipitation is 120–250 mm, and the January and July temperatures are −20 to-15 °C and 20–25 °C, respectively.
5. Semi-desert zone: This zone is approximately 1000–1500 m above sea level with warm and dry winters and hot and dry summers and an average precipitation of 50–150 mm.
6. Desert zone: The Gobi Desert is a cold desert with frost and occasional snow on its dunes. Besides being located far north, it is also located on a plateau approximately 910–1520 m above sea level, contributing to its low temperatures. It has an average rainfall of approximately 194 mm. Moreover, as the Siberian Steppes wind blows snow, additional moisture reaches parts of the Gobi in winter. These winds may cause the temperature in the Gobi Desert to reach −40 °C in winter and + 45 °C in summer. Furthermore, it has an extreme climate, combined with rapid temperature changes of as much as 35 °C.

### 2.2 Study population

This was a cross-sectional study involving individuals who experienced tick bites. The investigations were performed in the central hospital and Center for Zoonotic Diseases of each province in Mongolia.

The inclusion criteria included individuals who had been bitten by ticks or were suspected of being bitten by a tick; those who had not donated blood or plasma in the month prior to booster vaccination; and those who were not pregnant. All participants or their parents and guardians consented to participate in the TBE seroprevalence study. The exclusion criteria included participants who had not been bitten by ticks or were not suspected of being bitten by a tick, those who had given blood or plasma within one month before the booster vaccination, those who were pregnant, and participants and their parents and guardians who did not consent to participate in this study. The screened individuals were questioned regarding their TBEV vaccination status, including whether they had received the first, second, or third dose. If they had been vaccinated against TBEV and other flaviviruses, they were excluded. Individuals who did not respond to, receive, or complete the questionnaire were also excluded.

The survey was conducted between April 2016 and October 2022 during the seasonal activity of ticks. The study sample comprised 52% males (514/993) and 48% females (475/993), with an age range of 0–75 years [median age, 28 ± 20 years (0.85)].

### 2.3 Questionnaire

All participants were informed verbally and in writing regarding the investigation, including the voluntary and anonymous nature of participation, using an explanatory flyer approved by the ethics committee. The participants were interviewed using a paper-based questionnaire to collect data regarding demographic characteristics, sex, age, occupation status, exposure to ticks, previous tick-borne infections, and certain risk factors of TBE. For those under 18 years, the parents or guardians were required to answer the questionnaire. The completed questionnaires were collected anonymously.

### 2.4 Serology

After the interview, blood samples (10 mL) were collected from all patients in vacutainers (Horiba Medical®, Audicom Medical Instruments Co., Ltd. Jiangsu, China). Next, the serum was separated within 12 h and stored at −80 °C for future serological studies. Subsequently, enzyme-linked immunosorbent assay (ELISA) tests were conducted using the Euroimmun ELISA kit (Euroimmun AG, Medizinische Labor Diagnostika, Germany) as per the manufacturer's instructions. For the purposes of this study, a serum sample analysis was conducted to determine the presence of immunoglobulin (Ig) M and IgG antibodies specific to TBEV. Afterward, the optical density of the ELISA plates was read using an ELISA plate reader (Infinite® F50, Tecan Group Ltd., Männedorf, Switzerland) at 450 and 620 nm according to the manufacturer's instructions. The results were calculated and presented as recommended by the manufacturing company (Euroimmun AG, Medizinische Labor Diagnostika, Germany). Furthermore, antibody indices were calculated by dividing the test sample's odds ratio (OR) by the cut off calibrators' average OR (provided with the positive and negative serum discrimination kits). For the assay to be considered valid, patients with <0.8 were considered negative, and those with ≥1.1 were defined as patients who had contact with the antigen and, therefore, were positive for TBEV infection. The following criteria must be determined according to the manufacturer's instructions (Euroimmun AG, Medizinische Labor Diagnostika, Germany). Lastly, serum samples >0.8 and ≤ 1.1 were considered ambiguous and subjected to repeated testing. If the repeat result was ambiguous for a given sample, it was considered negative.

### 2.5 Statistics

All data were analyzed using the R software (the R Foundation for Statistical Computing, Vienna, Austria) (R.C. Team, 2013; Sjoberg et al., 2021); Wilcoxon rank sum test was used for within-group comparisons, Pearson's chi-squared test was used for testing between categorical responses, and Fisher's exact test was used to compare the sex and age groups. For age trend analysis, we used generalized additive (mixed) models built on R software using the mgcv package (Wood et al., 2017). Generalized additive models with thin lamellar regression splines were used to model and plot the functional relationship between age and seroprevalence of TBE in the study population. Next, age trends were presented for the entire study population and stratified by sex and year.

The seroprevalence of TBEV antibodies is associated with age and calendar period; therefore, age group and study year were included in the risk factor analysis. On binary logistic regression, age, and study year showed strong associations with TBEV seroprevalence. Furthermore, crude and adjusted OR values were estimated for age, sex, occupation, and the province of residence.

### 2.6 Ethics

This study was preformed according to the Helsinki Declaration and approved by the Medical Ethical Committee of the Mongolian National University of Medical Sciences (MEC No. 18–02/2A). Printed knowledgeable consent was obtained from all participants, who could withdraw their consent at any time.

## 3 Results

### 3.1 Seroprevalence analysis

**Fig. 1:**
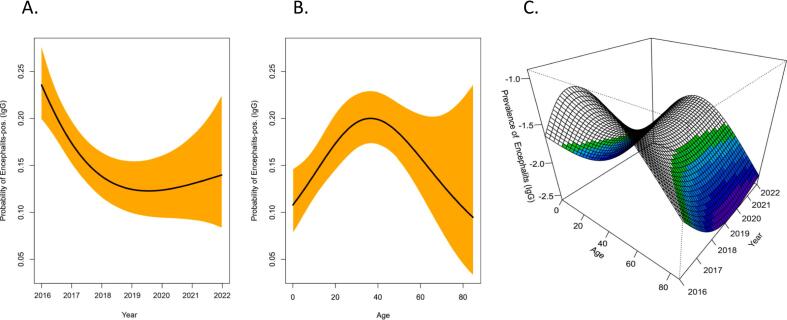
Overall TBEV seroprevalence trends by age. TBEV seroprevalence is plotted according to generalized additive model trend curves, with 95% confidence intervals assessed from non parametrically cluster bootstrapping 1000 by year (A) and age (B). Three-dimensional age-specific contour maps for TBEV seroprevalence probability for the target year (C). TBEV, tick-borne encephalitis virus.

**Fig. 2:**
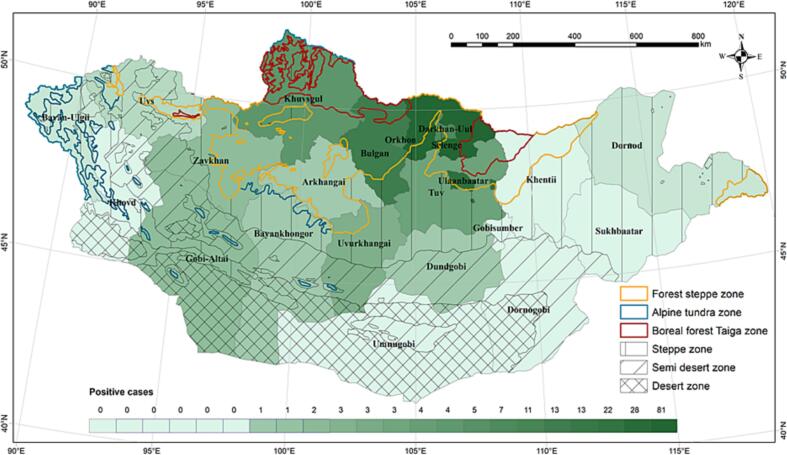
Spatial distribution of seroprevalence of TBEV in the participants. The color of each province corresponds to the seroprevalence of TBEV (darker colors indicate a higher seroprevalence, as shown in the legend). TBEV, tick-borne encephalitis virus.

TBEV IgM was identified in 8.1% of the serum samples (80/993). Additionally, 201serum samples (20.2%) were identified as positive for anti-TBEV IgG (Table 1). Seroprevalence of the anti-TBEV IgG antibody decreased gradually from 2016 to 2018 and then increased slowly from 2019 to 2022 (Fig. 2A). The TBEV seroprevalence significantly increased between 2016 and 2022 (*p* < 0.001), primarily driven by the high number of cases in 2017 (35%, 95% confidence interval [CI]: 0.31–0.73, OR: 0.48) and the low seroprevalence in 2021 (2.0%, 95% CI: 0.08–0.78) (Table 1, Fig. 1A). Lastly, TBEV was detected in Bayan-Ulgii and Gobi-Altai provinces for the first time using anti-TBEV IgM ELISA (1.3% and 1.3%, respectively) and anti-TBEV IgG ELISA (0.5% and 2.0%, respectively).

**Table 1:** Stratified seroprevalence of IgM and IgG antibodies against TBEV detected by ELISA in participants from 2016 to 2022, Mongolia.

Year	Encephalitis IgM			OR	95%CI		p-value^1^	Encephalitis IgG			OR	95%CI		p-value^1^
	Overall, N = 993	Number of negative samples *N* = 913 (92%)	Number of positive samples *N* = 80 (8.1%)		Lower	Upper		Overall, N = 993	Number of negative samples *N* = 792 (80%)	Number of positive samples *N* = 201 (20%)		Lower	Upper	
							<0.001							<0.001
2016	156 (16%)	153 (17%)	3 (3.8%)					156 (16%)	103 (13%)	53 (26%)				
2017	356 (36%)	323 (35%)	33 (41%)	5.21	1.83	21.9		356 (36%)	286 (36%)	70 (35%)	0.48	0.31	0.73	
2018	195 (20%)	185 (20%)	10 (12%)	2.76	0.83	12.5		195 (20%)	162 (20%)	33 (16%)	0.40	0.24	0.65	
2019	104 (10%)	95 (10%)	9 (11%)	4.83	1.40	22.2		104 (10%)	87 (11%)	17 (8.5%)	0.38	0.20	0.69	
2020	101 (10%)	82 (9.0%)	19 (24%)	11.8	3.89	51.4		101 (10%)	85 (11%)	16 (8.0%)	0.37	0.19	0.67	
2021	31 (3.1%)	28 (3.1%)	3 (3.8%)	5.46	0.97	30.9		31 (3.1%)	27 (3.4%)	4 (2.0%)	0.29	0.08	0.78	
2022	50 (5.0%)	47 (5.1%)	3 (3.8%)	3.26	0.59	18.1		50 (5.0%)	42 (5.3%)	8 (4.0%)	0.37	0.15	0.81	

1 Wilcoxon rank sum test; Pearson's Chi-squared test; Fisher's exact test.

### 3.2 Age trend analysis

TBEV seroprevalence was significantly higher among those aged 25–44 years than in those aged 60–75 years (p < 0.001) (Table 2). The seroprevalence of TBEV was related nonlinearly to the age of all the analyzed participants (Fig. 2). From age 0–4 to 35–39 years, TBEV seroprevalence increased gradually (Fig. 1B). A maximum TBEV seroprevalence of 11% was observed between the ages of 35 and 39 years, followed by a gradual decrease in seroprevalence from 10% to 2.0% between the ages of 40 and 69 years (Fig. 1C).

**Table 2:** Stratified seroprevalence of IgG antibodies against TBEV detected by ELISA in participants aged 0–75 years and results of weighted binary logistic regression analysis of potential risk factors for seropositivity, 2016–2022, Mongolia.

Characteristic	Overall, N = 993^1^	Number of negative sample N = 792 (80%)^1^	Number of positive sample N = 201 (20%)^1^	OR	95% CI		p-value^2^
					Lower	Upper	
Sex							<0.001
Female	475 (48%)	410 (52%)	65 (32%)				
Male	514 (52%)	378 (48%)	136 (68%)	2.25	1.63	3.13	
Occupation							<0.001
Self-employment	97 (9.8%)	63 (8.0%)	34 (17%)				
Employment /government, office, private sector/	204 (21%)	161 (20%)	43 (22%)	0.97	0.63	1.48	
Student /pupils/high schoolers, students/	229 (23%)	196 (25%)	33 (17%)	0.61	0.38	0.95	
Herders	139 (14%)	118 (15%)	21 (11%)	0.65	0.37	1.09	
Unemployment/at home, pension/	324 (33%)	254 (32%)	70 (35%)	0.51	0.31	0.84	
Median age	28 ± 20 (0.11, 85)	28 ± 20 (0.11, 85)	31 ± 18 (0.11, 66)	1.01	1	1.02	<0.04
Age group							<0.001
0–4	106 (11%)	87 (11%)	19 (9.5%)	–	–		
5–9	170 (17%)	154 (19%)	16 (8.0%)	0.48	0.23	0.97	
10–14	49 (4.9%)	38 (4.8%)	11 (5.5%)	1.33	0.56	3.02	
15–19	46 (4.6%)	40 (5.1%)	6 (3.0%)	0.69	0.24	1.77	
20–24	47 (4.7%)	38 (4.8%)	9 (4.5%)	1.08	0.43	2.56	
25–29	60 (6.0%)	38 (4.8%)	22 (11%)	2.65	1.29	5.51	
30–34	88 (8.9%)	67 (8.5%)	21 (10%)	1.44	0.71	2.9	
35–39	75 (7.6%)	53 (6.7%)	22 (11%)	1.9	0.94	3.87	
40–44	96 (9.7%)	75 (9.5%)	21 (10%)	1.28	0.64	2.58	
45–49	75 (7.6%)	55 (6.9%)	20 (10.0%)	1.67	0.82	3.42	
50–54	52 (5.2%)	41 (5.2%)	11 (5.5%)	1.23	0.52	2.79	
55–59	71 (7.2%)	60 (7.6%)	11 (5.5%)	0.84	0.36	1.87	
60–64	28 (2.8%)	20 (2.5%)	8 (4.0%)	1.83	0.68	4.69	
65–69	14 (1.4%)	10 (1.3%)	4 (2.0%)	1.83	0.46	6.15	
70–75	16 (1.6%)	16 (2.0%)	0 (0%)	0			
Residential area							<0.001
Arkhangai	31 (3.1%)	28 (3.5%)	3 (1.5%)	–	–		
Bayan-Ulgii	8 (0.8%)	7 (0.9%)	1 (0.5%)	1.33	0.06	12.4	
Bayankhongor	29 (2.9%)	26 (3.3%)	3 (1.5%)	1.08	0.18	6.28	
Bulgan	86 (8.7%)	64 (8.1%)	22 (11%)	3.11	0.98	13.9	
Darkhan-Uul	55 (5.5%)	42 (5.3%)	13 (6.5%)	2.89	0.84	13.4	
Dornod	14 (1.4%)	13 (1.6%)	1 (0.5%)	0.72	0.03	6.24	
Dornogobi	3 (0.3%)	3 (0.4%)	0 (0%)	0			
Dundgobi	9 (0.9%)	6 (0.8%)	3 (1.5%)	4.67	0.72	31.4	
Gobi-Altai	39 (3.9%)	35 (4.4%)	4 (2.0%)	1.07	0.22	5.79	
Gobisumber	3 (0.3%)	3 (0.4%)	0 (0%)	0			
Khentii	14 (1.4%)	14 (1.8%)	0 (0%)	0	0.00	0.00	
Khovd	20 (2.0%)	20 (2.5%)	0 (0%)	0	0.00	0.00	
Khuvsgul	69 (6.9%)	58 (7.3%)	11 (5.5%)	1.77	0.50	8.28	
Orkhon	54 (5.4%)	41 (5.2%)	13 (6.5%)	2.96	0.86	13.8	
Selenge	230 (23%)	149 (19%)	81 (40%)	5.07	1.73	21.7	
Sukhbaatar	1 (0.1%)	1 (0.1%)	0 (0%)	0			
Tuv	19 (1.9%)	12 (1.5%)	7 (3.5%)	5.44	1.28	28.8	
Ulaanbaatar city	194 (20%)	166 (21%)	28 (14%)	1.57	0.51	6.89	
Umnugobi	9 (0.9%)	9 (1.1%)	0 (0%)	0	0.00	0.00	
Uvs	37 (3.7%)	35 (4.4%)	2 (1.0%)	0.53	0.07	3.43	
Uvurkhangai	31 (3.1%)	26 (3.3%)	5 (2.5%)	1.79	0.40	9.46	
Zavkhan	36 (3.6%)	32 (4.0%)	4 (2.0%)	1.17	0.24	6.35	

1 Median ± SD (Range); n (%)

2 Wilcoxon rank sum test; Pearson's Chi-squared test; Fisher's exact test.

To investigate potential synergistic statistical interactions between age and TBEV seroprevalence, without casually categorizing either continuous variable, generalized additive models that used two-dimensional smoothing splines for age and year were used to generate a graph of the three-dimensional association (Fig. 1). The association of age with TBEV seroprevalence was retained across all target years in all participants from the trend analysis (Fig. 1B). The steep slope of the relationship between age and seroprevalence, relative to the relationship between year and seroprevalence, implied that age was related to TBEV seroprevalence rather than to the year (similar to the risk factor analysis results below). Further, the relationship between year and TBEV seroprevalence was virtually nonlinear. The relationship between age and year, which synergistically increased (or decreased) the conditional probability of TBEV infection, appeared on the three-dimensional risk surface.

### 3.3 Risk factor analysis

Binary logistic regression analysis was performed to assess the potential risk factors for TBEV among participants with IgG-positive serum samples. TBEV seroprevalence was significantly higher in males (68%, 95% CI: 1.63–3.13, OR: 2.25) than in females (*p* < 0.001) and significantly higher among unemployed (35.0%, 95% CI: 0.31–0.84, OR: 0.51) than employed participants. Furthermore, the prevalence was the highest among participants aged 25–29 and 35–39 years (11%, 95% CI: 1.29–5.51, OR: 2.65; 11%, 95% CI: 0.94–3.87, OR: 1.9, respectively), and the lowest among those aged 65–69 years (2%, 95% CI: 0.46–6.15, OR: 1.83). TBEV seroprevalence was 9.5% and 8.0% in those aged 0–4 and 5–9 years, respectively. Therefore, TBEV seroprevalence was significantly different among the age groups (*p* < 0.001). Lastly, according to the univariate subgroup analysis, age, occupation, and residential area were significantly associated with TBEV seroprevalence (Table 2).

### 3.4 Spatial distribution

TBEV seroprevalence was mapped according to province (Fig. 2), and we collected serum samples from all 21 provinces and the capital city Ulaanbaatar. TBEV was detected in 15 provinces and Ulaanbaatar, with the highest seroprevalence in Selenge province and Ulaanbaatar (40%, 95% CI: 1.73–21.7, OR: 5.07;14%, 95% CI: 0.51–6.89, OR: 1.57, respectively) and the lowest in Bayan-Ulgii and Dornod provinces (0.5%, 95% CI: 0.06–12.4, OR: 1.33; 0.5%, 95% CI: 0.03–6.24, OR: 0.72, respectively). Lastly, TBEV seroprevalence was significantly different among the provinces (p < 0.001) (Table 2). The TBEV seroprevalence estimates for each neighborhood were mapped for the study population. The shade of each neighborhood corresponds to its TBEV seroprevalence (darker shades correspond to higher seroprevalence). Neighborhoods shaded in gray represent those in which the point estimate was statistically inconsistent because of a small sample size (Fig. 2).

## 4 Discussion

Using ELISA, we analyzed 993 serum samples collected from 21 Mongolian provinces and the capital city Ulaanbaatar. The TBEV seroprevalence was 20.2% (201/993) in 15 provinces and Ulaanbaatar between 2016 and 2022. According to the epidemiological surveillance of the population between 1998 and 2004, 12.5% of the participants (153/1221) from 20 districts of 7 provinces in Mongolia had detectable TBEV antibodies (Bata et al., 2005; Bataa et al., 2005). Furthermore, between 1998 and 2005, 161 tick-borne infections and 90 TBE cases were linked to TBEV (Adiyasuren et al., 2002), and between 2005 and 2016, neutralizing antibodies against TBEV were found in 14.6 ± 7.3% of the population from 47 districts in 10 provinces and Ulaanbaatar (Baasandavga et al., 2017). These results show that the infection incidence and prevalence of TBEV in humans are increasing in Mongolia. From our study, we concluded that the incidence of TBEV infection remains low in most regions of Mongolia but is increasing in endemic areas. Therefore, besides Mongolia (Adiyasuren et al., 2002; Bata et al., 2005; Bataa et al., 2005), TBEV infection is an increasing public health burden in Russia (Ruzek et al., 2019), China (Chen et al., 2019), Central Europe (Heinz et al., 2013), and South-east Asia (Pommier et al., 2022), and it can be greatly reduced by vaccination (Baasandavga et al., 2019; Steffen, 2016; Schmidt et al., 2022).

TBE is diagnosed by testing for specific antibodies (Holzmann, 2003). The serological analysis of TBEV disease is based on the positive findings for IgM or IgG antibodies. However, during the first phase and initiation of the second phase or monophasic course, the serological analysis results may be negative. Therefore, the test must be repeated after a few days (Ruzek et al., 2019). In this study, we conducted first- and second-phase serological surveys. Serum IgM antibodies typically develop within 3–7 days after infection and function as TBEV impact markers. Moreover, serum IgG antibodies appear within 10–14 days and can persist for many years (Kaiser and Holzmann, 2000). In this study, we presented a seroprevalence analysis based on IgM and IgG findings. However, the trends in age, risk factors, and spatial distribution were assessed based solely on IgG findings.

In this study, significantly more men (68%) than women (*p* < 0.001) were affected. According to the literature, men are affected more frequently than women in all age groups, and the highest TBEV infection rate was observed in individuals aged 45–64 years, followed by individuals >65 years (Logar et al., 2006). Furthermore, in European countries, the highest TBEV infection rate was reported among those aged 60–69 years, reflecting the high exposure of this age group to ticks, considering that this age group has more time for outdoor recreation and is prone to high disease severity (Beauté et al., 2018; Lindquist, 2014). However, in our study, the TBEV infection rate was the lowest in those aged 60–69 and the highest in individuals aged 25–29 and 35–39 years. This result may be attributed to the following reasons. First, older individuals may have greater awareness and knowledge regarding tick-borne diseases and may be more cautious when engaging in outdoor activities. Additionally, their exposure to the outdoors may be lower than that of younger individuals, reducing the likelihood of tick bites. The higher infection rate among younger individuals may be due to their increased outdoor activity and exposure to tick habitats as well as a potential lack of awareness and preventive measures. Overall, age may play an important role in TBEV infection rates and should be considered when developing preventive strategies. Furthermore, our study showed that males had a higher infection rate compared to females, which is consistent with previous research. This may be due to differences in behavior and occupational activities, as men tend to spend more time outdoors engaging in activities, such as hunting, fishing, herding, and forestry work. In addition, hormonal differences between males and females may also affect their susceptibility to tick-borne diseases. Geographic location was another important factor related to TBEV infection rates, with individuals living in rural areas having a higher infection rate than those in urban areas. This may be due to differences in tick habitat and exposure and a lack of awareness and preventive measures in rural areas. Additionally, individuals who travel frequently to and from tick-endemic areas may also be at a higher risk for infection. Our study highlights the importance of education and preventive measures for tick-borne diseases and the need to consider demographic and geographic factors when developing strategies to control and prevent these infections. Raising awareness and promoting preventive measures could help reduce the incidence of tick-borne diseases and improve overall public health.

At the end of 2020, 608,090 Mongolians were actively seeking work; this group included higher proportions of individuals aged 25–34 (35.6%) and 35–44 (25.6%) years than of those aged 45–54 and 55–59 years. Moreover, Mongolia had the highest youth unemployment rate (19.2%) in the Asia-Pacific region in 2018, even higher than the global average (Mongolian Statistical Information Service, 2021, 2022). Unemployed Mongolian young adults are more likely to spend time outdoors engaging in activities, such as gold mining or ninja mining (High, 2007; Munkherdene and Sneath, 2018), working in forests to collect wood, herding, and hunting in endemic areas where there is a high risk of tick bites. According to the WHO, the primary mode of TBEV transmission is through the bite of an infected tick (World Health Organization, 2020b). The severity of TBE increases with age, resulting in more cases of encephalitis related to meningitis among older individuals (George et al., 2014). Independent of undefined age-specific physiologic characteristics that control disease severity, the risk for virus exposure might differ among age groups depending on behavioral factors. Analysis of the age distribution of patients with TBE in the Czech Republic, Slovenia, and Austria during 1990–1999 and 2000–2010 showed significant differences. Incidence rates increased sharply among those aged 50–80 years in the Czech Republic and Slovenia. TBE incidence among children and adolescents, which is substantial in Slovenia and the Czech Republic, has virtually disappeared in Austria (Heinz et al., 2013). In the current study, the TBEV seroprevalence rates were 9.5% and 8.0% in those aged 0–4 and 5–9 years, respectively. Therefore, efforts must be made to prevent and control of TBEV transmission in this young population. In this study, the youngest female patient infected with TBEV was 11 months old. It is unclear how children aged 0–4 years are exposed to TBEV. However, these findings may be attributable to older individuals having developed immunity due to previous exposures to similar viruses and being more likely to take preventive measures to avoid TBEV exposure. The higher prevalence rate in endemic areas among nomadic people and their children might be related to their lifestyle as they spend most of their time outdoors (Myadagsuren et al., 2007). Lastly, an average of 10–20% of all reported TBEV infection cases were reported in children, with the lowest frequency in children <3 years (Fowler et al., 2013; Krawczuk et al., 2020; Arnez and Avsic-Zupanc, 2009).

TBEV infection is endemic to Mongolia (Frey et al., 2012; Baasandavga et al., 2019), especially to Selenge and Bulgan provinces, which have a high prevalence of TBEV (Bataa et al., 2005). In a previous study, most patients recalled tick bites occurring in the Selenge (78%) or Bulgan (12%) province (Baasandavga et al., 2017), which is consistent with the findings of our study, according to which tick bites mostly occurred in the Selenge (23%) and Bulgan (8.7%) regions. Furthermore, *I. persulcatus* is the primary vector of TBEV in Mongolia. Selenge province was found to have the highest prevalence of *I. persulcatus* ticks carrying TBEV (Muto et al., 2015; Khasnatinov et al., 2010c; Boldbaatar et al., 2017).Therefore, most parts of Selenge and Bulgan provinces are high-risk TBEV infection areas (Černý et al., 2019).This may be the primary reason for the high TBEV seroprevalence in Selenge (40%) and Bulgan provinces (11%) compared with that in other provinces between 2016 and 2022. Furthermore, vaccination and education interventions are mainly concentrated in the Selenge and Bulgan provinces, which have the largest population of *I. persulcatus* and arethe Taiga-covered parts of Mongolia (Muto et al., 2015; Boldbaatar et al., 2017; Lagunova et al., 2022). This result shows that Selenge and Bulgan provinces are at a higher risk than other provinces. *Ixodes* ticks, which are the primary carriers of TBEV, have been observed in various regions across Mongolia, including Selenge, Bulgan, Orkhon, Tuv, Khentii, and Dornod provinces. Studies indicate that approximately 3.4% of these ticks carry the TBEV (Frey et al., 2012; Damdindorj et al., 2021; Bata et al., 2005; Bataa et al., 2005; Černý et al., 2019). Furthermore, *Dermacenter* ticks not only act as vectors but also serve as effective reservoir hosts for carrying viruses (Boldbaatar et al., 2017; Černý et al., 2019; Narankhajid et al., 2018).

Serological surveys should be conducted to identify other potential cases and their related contacts. Furthermore, the situation should be continuously monitored to advise residents and national and foreign travelers to take extra precautions and prevent the spread of TBEV infection.

In our study, Dornod province (steppe biogeographical zone) had 0.5% of the TBEV-positive cases and Dundgobi province (desert biogeographical zone) had 1.5%. Besides *I. persulcatus,* the predominant TBEV vectors in Mongolia include *Dermacentor silvarum* and *D. nuttalli (*Baasandavga et al., *2017**)*. *D. nuttalli* is a tick species distributed nationwide across all biogeographical zones in Mongolia (Narankhajid et al., 2018).

The epidemic curve of the highest TBEV seroprevalence in Mongolia, with sizable peak case numbers observed in 2016 (26%) and 2017 (35%), has not been sufficiently elucidated. These peaks might have resulted because of poverty following the economic downturn, resulting in many unemployed people taking part in a gold rush (ninja miner) and collecting woods to survive. Furthermore, in the first documented human case of TBE in 1989, the natural foci were observed in the Bugant, Khuder, and Zelter districts of Selenge province (Damdindorj et al., 2021), possibly because these regions are the primary areas for gold mining by ninja miners. This high incidence of TBEV infection in Mongolia can be attributed to the following issues: (1) The risk of tick bites has been increasing with the increasing unemployed population. Additionally, ninja miners in particular areas have been infected with TBEV. However, it is questionable whether endemic foci of TBEV are situated in areas where people, predominantly ninja miners, and emergency and forest workers, are infected easily without fluctuations in the case numbers. Therefore, there is an urgent need for serological surveys among residents of TBEV-endemic areas in Mongolia to establish adequate precautionary procedures. (2) Furthermore, in several subarctic countries, an increase in TBEV infection incidence has been observed in recent decades (Lindgren and Gustafson, 2001; Lukan et al., 2010; Tokarevich et al., 2011). Without excluding other factors, some researchers have concluded that climate change has largely contributed to increasing TBEV infection incidence (Danielová et al., 2008; Tokarevich et al., 2011; Gray et al., 2009). (3) In tick-active months, herders hire unemployed people, high school pupils, and students to help care for domestic animals and comb-out goats. Consequently, these groups are likely to be infected with TBEV. Additionally, the local natural risk is largely decided by the density and movement of the infected ticks (Süss et al., 2004; Kahl, 1996). This, together with human exposure (traveling to or visiting endemic areas or local natural foci), increases the risk of host infection. Furthermore, a person's behavior can significantly affect the risk of TBEV infection, such as being completely exposed to ticks or avoiding tick-infested areas in natural foci, using effective tick-repellant products, and receiving vaccination.

Our study has some limitations. First, only a few individuals from some provinces participated in the study. Second, some participants did not complete the questionnaire; hence, some data were missing. For the purpose of this study, only those participants who completed the questionnaire were included for the analysis. Third, we did not collect sufficient data during the COVID-19 pandemic, as Mongolia was under complete and partial quarantine between 2021 and 2022.

## 5 Conclusion

Overall, TBEV seroprevalence has increased in endemic areas; however, it is low in non-endemic areas. Moreover, TBEV was detected in Bayan-Ulgii and Gobi-Altai provinces for the first time between 2016 and 2022. Furthermore, surveillance at the national level has helped generate reliable and comparable data to map disease risk at national and provincial levels. Therefore, in provinces where the disease is highly endemic, information campaigns on preventive procedures against tick bites should be conducted and TBE vaccination recommendations should be provided.

## Declaration of Competing Interest

The authors declare that they had no conflicts of interest with respect to their authorship or the publication of this article.

Submitted to the Parasite Epidemiology and Control.
